# Concerns with the Usage of ChatGPT in Academia and Medicine: A Viewpoint

**DOI:** 10.1016/j.ajmo.2023.100036

**Published:** 2023-02-27

**Authors:** Muath Alser, Ethan Waisberg

**Affiliations:** a Cairo University Faculty of Medicine, Cairo, Egypt; b University College Dublin School of Medicine, Belfield, Dublin, Ireland

We have written this article to express our concerns about the growing trends of ChatGPT usage in academia and medicine. ChatGPT is an artificial intelligence large language model developed by OpenAI and launched on 30th November 2022 as a free beta version in many countries worldwide. This language model can produce human-like text responses to users’ prompts in almost any field.

## ChatGPT Authorship

Recently, 2 articles have been published in peer-reviewed medical journals and 2 other preprints where ChatGPT was credited authorship.^1^, ^2^, ^3^, ^4^

As per the International Committee of Medical Journal Editors (ICMJE) guidelines for authorship, to be eligible for authorship, you need to fulfill all these 4 criteria^5^:

1. “Substantial contributions to the conception or design of the work; or the acquisition, analysis, or interpretation of data for the work; AND
2. Drafting the work or revising it critically for important intellectual content; AND
3. Final approval of the version to be published; AND
4. Agreement to be accountable for all aspects of the work in ensuring that questions related to the accuracy or integrity of any part of the work are appropriately investigated and resolved.”^5^

In the case of ChatGPT authorship, in 3 out of the 4 examined papers, ChatGPT made a substantial contribution in writing parts of the paper (only 1 criterion applies),^1,^^2,^^4^ while in the remaining paper, ChatGPT's answers were further analyzed by human authors, so ChatGPT served as a research participant, not an author (no criteria applies).^3^ In all of these papers, ChatGPT did not approve the final version of the published article, nor did it agree to be responsible for the publication's accuracy or declare any conflict of interest. And even if the chatbot approved these publications, should we consider its approval legally significant?

## ChatGPT Plagiarism

We performed a plagiarism check on the parts of writing contributed by ChatGPT in 1 of these articles using Grammarly, Plagiarism Checker X, and PlagScan (by Turnitin). Unsurprisingly, plagiarism was detected after manual review, ranging from 5% to 33.8% and reaching 48.9% after merging all of the scans (see supplementary file).

Some phrases detected by these tools were copied word for word by ChatGPT from unreliable sources like Wikipedia, LinkedIn, Medium, and even the Apple App Store (direct plagiarism).

ChatGPT did not provide by default any citations for its output (paraphrasing plagiarism). When asked for references for some of its answers, it provided citations that do not exist (source-based plagiarism). When asking ChatGPT the same prompt again, it gives very similar answers, which could lead to self-plagiarism.

Including ChatGPT's output as it is in the manuscript without quotation marks gives the impression that this part is contributed by all authors (misleading attribution). Citing ChatGPT for its output is unethical, too, as the primary source of information is not cited (source-based plagiarism). Hence plagiarism is inevitable when using ChatGPT.

## ChatGPT Sources of Bias

Up to this point, OpenAI has not published the source code for ChatGPT, nor has it explained its learning data sources. Asking ChatGPT about its data updates, it mentioned that its knowledge is limited to 2021, which means that it is outdated for 2023.

Based on our plagiarism checks, ChatGPT uses academic and nonacademic sources and apparently does not differentiate between sources of information based on their level of evidence. This accounts for the errors that ChatGPT can occasionally make.

Developers have fine-tuned the output of ChatGPT through supervised and reinforcement learning (reward system for desired answers); this could bias the output toward the developers’ opinions. More seriously, public users can participate in this tuning through upvoting or downvoting answers; this manipulation devalues the output from a scientific point of view.

## ChatGPT in Medical Education and Clinical Practice

One of the aforementioned preprints that gave authorship to ChatGPT suggested the use of language models in medical education and in leading clinical decisions as the chatbot passed a USMLE exam!^3^ Many of the USMLE exams are publicly available online with answers. The passing of USMLE by this bot reflects its ability as a search engine rather than as a reliable source for clinical decisions.

This hypothesis is supported by a Korean study that compared ChatGPT's performance in a parasitology exam with medical students’ performance and found that ChatGPT scored significantly less than the medical students.^6^

## Final Remarks

ChatGPT is a language model that analyzes statistical patterns of language used throughout a large data set. This makes this bot capable of producing appealing writings language-wise, but it lacks depth and factual accuracy. Due to the previously explained serious drawbacks of this ChatGPT version, we should recommend against its usage in academia. In case of the unavoidable need for the use of this bot in scientific publications, we should acknowledge the bot, but not grant authorship, while paying attention to different types of plagiarism and biases it has.

## Declaration of Competing Interest

The authors declare that they have no conflict of interest.

## Appendix Supplementary materials

Image, application 1
