# Supplementary material for: Concerns with the Usage of ChatGPT in Academia and Medicine: A Viewpoint
Source: Am J Med Open. 2023 Feb 27;9:100036. doi: 10.1016/j.ajmo.2023.100036 (PMC11256230; doi:10.1016/j.ajmo.2023.100036)
Supplement: Supplementary file 1 [file mmc1.pdf]

Supplementary file: Summary of plagiarism reports

"Artificial intelligence (AI) is the simulation of human intelligence in machines that are programmed to think and act like humans. These machines are designed to learn, reason, and solve problems in a way that is like human cognition, and they can perform tasks that typically require human intelligence, such as understanding language, recognizing images, and making decisions. The goal of AI research is to create systems that are capable of intelligent behaviour and can be used to improve various aspects of our lives, from healthcare and education to transportation and entertainment. An AI chatbot is a type of chatbot that uses artificial intelligence to enable it to understand and respond to user input in a natural, human-like way. AI chatbots are designed to have conversations with people, using natural language processing to understand and respond to the user's words and intentions. They can be used in a variety of settings, such as customer service, where they can provide answers to common questions and help customers resolve issues, or as personal assistants, where they can help users manage their schedules and tasks. Some AI chatbots are also used in entertainment, such as in video games or as virtual companions.

AI chatbots can be used in education to provide personalized learning experiences for students. For example, an AI chatbot could be used to help students learn a new language by engaging them in conversation and providing feedback and corrections. AI chatbots could also be used to provide tutoring and homework help, by answering questions and providing explanations to help students understand difficult concepts. In addition, AI chatbots could be used to help students stay organized and manage their time, by providing reminders and helping them schedule their tasks and assignments. Overall, AI chatbots have the potential to enhance education by providing personalized, interactive learning experiences for students.

Plagiarism is the act of using someone else's work without proper attribution, and it is a serious issue in education. AI chatbots can help prevent plagiarism by providing students with tools and resources that can help them properly cite and reference their sources. For example, an AI chatbot could be programmed to scan a student's paper for common instances of plagiarism, such as copied text or unattributed ideas, and provide suggestions for how to properly attribute the source. In addition, an AI chatbot could provide students with information and guidance on how to avoid plagiarism, such as by providing tips on paraphrasing and summarizing, and by explaining the importance of proper citation and attribution.

However, it is possible that an AI chatbot could cause plagiarism if it is not used responsibly and ethically. For example, if an AI chatbot provides students with information or answers without properly citing the source, it could inadvertently lead students to plagiarize. In addition, if an AI chatbot is programmed to generate text or content without properly attributing the source, it could produce work that is plagiarized. Therefore, it is important for the designers and developers of AI chatbots to carefully consider the potential risks of plagiarism and to take steps to prevent it. This may involve implementing safeguards and protocols to ensure that the information provided by the AI chatbot is properly attributed and that students are encouraged to properly cite and reference their sources. It may also involve educating students and teachers about the importance of academic integrity and the consequences of plagiarism. The potential use of artificial intelligence software by nursing students to write university assessments is a worrying possibility. AI software is designed to mimic human behaviour, but it is not designed to replace human creativity and critical thinking. As such, AI software cannot substitute for the knowledge, skills, and critical thinking that nursing students must develop and demonstrate in their university assessments. The use of AI software could lead to an erosion of the quality of the assessment, with implications for the development of the nursing profession. Furthermore, the use of AI software could lead to a lack of accountability among nursing students, as it is not possible to verify that the work submitted is their own. Finally, the use of AI software could lead to a decrease in the value of the assessments and the overall quality of the nursing program.

OpenAI is a research institute focused on developing artificial intelligence in a way that is safe and beneficial to humanity. The institute was founded in 2015 by Elon Musk and other prominent tech leaders, and its goal is to advance and promote AI research and development with the aim of creating advanced and intelligent technologies that can help improve the world. OpenAI is known for developing cutting-edge AI technologies and for conducting research in a variety of areas, including natural language processing, computer vision, and robotics. ChatGPT is a large language model trained by OpenAI. It is a machine learning system that is designed to be able to understand and generate natural human language in order to assist with a wide range of tasks, such as answering questions and providing information. It is not a human but is designed to be able to communicate and interact with people in a way that is similar to how a person would. It is constantly learning and improving, and there to help with whatever questions or information you need.” **(1)**

(1) O'Connor S, ChatGpt. Open artificial intelligence platforms in nursing education: Tools for academic progress or abuse? Nurse Educ Pract 2023;66:103537. DOI: 10.1016/j.nepr.2022.103537 [published Online First: 2022/12/23]

Table 1: summary of plagiarism reports to the previous writing produced by ChatGPT

**Plagiarism Check tool**	**Plagiarism score after manual check**	**Highlighted original sources for the direct plagiarism**	**Note**
Grammarly	9%	Medium, LinkedIn, Reverso, seminar	67 suggested corrections related to writing clarity, correctness, and originality
Plagiarism Checker X	12%	TechZone blog (7%), SmartClick blog (2%), Apple App Store, Wikipedia, CertificationAnswers, GraduateWay (database for essays)	-
PlagScan (Turnitin)	5.2%	Non-academic websites	Medium Sensitivity
PlagScan (Turnitin)	33.8%	Previously scanned documents, Patents website, CourseHero website, and other non-academic websites	High Sensitivity
Total	48.9%	Social media and non-academic websites (rarely from ScienceDirect)	(432/883) Counted all detected words by any tool/total word count

Please note that some highlighted phrases are overlapping between two plagiarism tools or more, for more details refer to the following reports for each tool separately.

Online Link to full PlagScan report:
https://www.plagscan.com/doc?148305726&sharekey=CDvqmrgEqDcdVyOncEFJ

33.8% PlagScan by Turnitin Results of plagiarism analysis from 22/01/2023, 01:28

ChatGPT2

Date: 22/01/2023, 01:27

✱ All sources 27 | Internet sources 8 | Own documents 1 | Plagiarism Prevention Pool 18

- [1] from a PlagScan document dated 2022-01-24 15:45
  25.5% 31 matches
- [2] from a PlagScan document dated 2022-01-25 11:26
  23.3% 31 matches
- [3] from a PlagScan document dated 2020-11-17 17:50
  19.0% 28 matches
- [4] from a PlagScan document dated 2022-06-07 10:37
  19.8% 29 matches
- [5] from a PlagScan document dated 2021-11-07 12:32
  17.0% 25 matches
- [6] from a PlagScan document dated 2019-09-14 12:24
  13.6% 26 matches
- [7] from a PlagScan document dated 2021-02-03 17:28
  10.2% 12 matches
- [8] from a PlagScan document dated 2019-05-16 07:22
  9.0% 16 matches
- [9] builtin.com/artificial-intelligence
  9.2% 14 matches
- [10] patents.justia.com/patent/10478719
  6.8% 17 matches
- [11] from a PlagScan document dated 2022-07-21 07:12
  6.9% 9 matches
- [12] from a PlagScan document dated 2021-01-14 09:58
  6.0% 8 matches
- [13] from a PlagScan document dated 2021-07-18 08:08
  5.6% 9 matches
- [14] www.nursingprocess.org/ethical-principles-in-nursing.html
  6.6% 10 matches
- [15] from a PlagScan document dated 2021-05-31 13:54
  4.9% 13 matches
- [16] from a PlagScan document dated 2018-05-28 04:02
  5.2% 13 matches
- [17] from a PlagScan document dated 2022-03-14 02:14
  4.2% 11 matches
- [18] from a PlagScan document dated 2022-06-16 15:27
  4.0% 13 matches
- [19] from a PlagScan document dated 2020-08-05 07:05
  4.0% 7 matches
- [20] becominghuman.ai/can-artificial-intelligence-be-at-par-with-or-even-surpass-human-intelligence-c2330e77d36c
  3.7% 10 matches
- [21] from a PlagScan document dated 2020-02-25 07:08
  3.5% 10 matches
- [22] youevolve.net/de/can-artificial-intelligence-replace-human-intelligence-2/
  3.3% 7 matches
- [23] from a PlagScan document dated 2018-05-12 04:59
  0.7% 3 matches
- [24] tr-ex.me/traduccion/inglés-español/cannot substitute for
  0.8% 2 matches
- [25] context.reverso.net/traduccion/ingles-espanol/cannot substitute
  0.6% 1 matches
- [26] www.coursehero.com/tutors-problems/Information-Security/46001563-Optus-have-been-in-the-media-for-the-last-couple-of-months-due-to-a/
  0.7% 1 matches

2 pages, 883 words

PlagLevel: 33.8% selected / 99.1% overall

35 matches from 27 sources, of which 8 are online sources.

Settings

Data policy: *Compare with web sources, Check against my documents, Check against the Plagiarism Prevention Pool*

Sensitivity: *High*

Bibliography: *Consider text*

Citation detection: *Highlighting only*

Whitelist: --

[9]Artificial intelligence (AI) is the simulation of human intelligence in machines that are programmed to think and act like humans.[3]These machines are designed to learn, reason, and solve problems in a way that is like human cognition, and they can perform tasks that typically require human intelligence, such as understanding language, recognizing images, and making decisions.[1]The goal of AI research is to create systems that are capable of intelligent behaviour and can be used to improve various aspects of our lives, from healthcare and education to transportation and entertainment.[6]An AI chatbot is a type of chatbot that uses artificial intelligence to enable it to understand and respond to user input in a natural, human-like way. [2]AI chatbots are designed to have conversations with people, using natural language processing to understand and respond to the user's words and intentions.[1]They can be used in a variety of settings, such as customer service, where they can provide answers to common questions and help customers resolve issues, or as personal assistants, where they can help users manage their schedules and tasks.[2]Some AI chatbots are also used in entertainment, such as in video games or as virtual companions.

[8]AI chatbots can be used in education to provide personalized learning experiences for students.[8]For example, an AI chatbot could be used to help students learn a new language by engaging them in conversation and providing feedback and corrections. [8]AI chatbots could also be used to provide tutoring and homework help, by answering questions and providing explanations to help students understand difficult concepts.[1]In addition, AI chatbots could be used to help students stay organized and manage their time, by providing reminders and helping them schedule their tasks and assignments.[10]Overall, AI chatbots have the potential to enhance education by providing personalized, interactive learning experiences for students.

[16]Plagiarism is the act of using someone else's work without proper attribution, and it is a serious issue in education.[4]AI chatbots can help prevent plagiarism by providing students with tools and resources that can help them properly cite and reference their sources.[4]For example, an AI chatbot could be programmed to scan a student's paper for common instances of plagiarism, such as copied text or unattributed ideas, and provide suggestions for how to properly attribute the source.[3]In addition, an AI chatbot could provide students with information and guidance on how to avoid plagiarism, such as by providing tips on paraphrasing and summarizing, and by explaining the importance of proper citation and attribution.

[3]However, it is possible that an AI chatbot could cause plagiarism if it is not used responsibly and ethically.[8]For example, if an AI chatbot provides students with information or answers without properly citing the source, it could inadvertently lead students to plagiarize.[10]In addition, if an AI chatbot is programmed to generate text or content without properly attributing the source, it could produce work that is plagiarized.[7]Therefore, it is important for the designers and developers of AI chatbots to carefully consider the potential risks of plagiarism and to take steps to prevent it.[1]This may involve implementing safeguards and protocols to ensure that the information provided by the AI chatbot is properly attributed and that students are encouraged to properly cite and reference their sources.[1]It may also involve educating students and teachers about the importance of academic integrity and the consequences of plagiarism.[1]The potential use of artificial intelligence software by nursing students to write university assessments is a worrying possibility.[1]AI software is designed to mimic human behaviour, but it is not designed to replace human creativity and critical thinking.[11]As such, AI software cannot substitute for the knowledge, skills, and critical thinking that nursing students must develop and demonstrate in their university assessments.[3]The use of AI software could lead to an erosion of the quality of the assessment, with implications for the development of the nursing profession.[7]Furthermore, the use of AI software could lead to a lack of accountability among nursing students, as it is not possible to verify that the work submitted is their own.[1]Finally, the use of AI software could lead to a

decrease in the value of the assessments and the overall quality of the nursing program.
[1]OpenAI is a research institute focused on developing artificial intelligence in a way that is safe and beneficial to humanity.[1]The institute was founded in 2015 by Elon Musk and other prominent tech leaders, and its goal is to advance and promote AI research and development with the aim of creating advanced and intelligent technologies that can help improve the world.[4]OpenAI is known for developing cutting-edge AI technologies and for conducting research in a variety of areas, including natural language processing, computer vision, and robotics.[1]ChatGPT is a large language model trained by OpenAI.[5]It is a machine learning system that is designed to be able to understand and generate natural human language in order to assist with a wide range of tasks, such as answering questions and providing information.[5]It is not a human but is designed to be able to communicate and interact with people in a way that is similar to how a person would.[1]It is constantly learning and improving, and there to help with whatever questions or information you need.

Plagiarism Checker X - Report

Originality Assessment

12%

Overall Similarity

Date: Jan 14, 2023
Matches: 109 / 883 words
Sources: 6

Remarks: Low similarity detected, check with your supervisor if changes are required.

Verify Report:
Scan this QR Code

[1] Artificial intelligence (AI) is the simulation of human intelligence in machines that are programmed to think and act like humans. These machines are designed to learn, reason, and solve problems in a way that is like human cognition, and they can perform tasks that typically require human intelligence, such as understanding language, recognizing images, and making decisions. The goal of AI research is to create systems that are capable of intelligent behaviour and [1] can be used to improve various aspects of our lives, from healthcare and education to transportation and entertainment. An AI chatbot [2] is a type of chatbot that uses artificial intelligence to enable it to understand and respond to user input in a natural, human-like way. AI chatbots are designed to have conversations with people, using natural language processing to understand and respond to the user's words and intentions. They can be used [5] in a variety of settings, such as customer service, where they can provide answers to common questions and help customers resolve issues, or as personal assistants, where they can help users manage their schedules and tasks. Some AI chatbots are also used in entertainment, such as in video games or as virtual companions.

AI chatbots can be used in education to provide personalized learning experiences for students. For example, an AI chatbot could [1] be used to help students learn a new language by engaging them in conversation and providing feedback and corrections. AI chatbots could also be used to provide tutoring and homework help, by answering questions and providing explanations to help students understand difficult concepts. In addition, AI chatbots could be used to help students stay organized and manage their time, by providing reminders and helping them schedule their tasks and assignments. Overall, AI chatbots have the potential to enhance education by providing personalized, interactive learning experiences for students.

Plagiarism is the act of using someone else's work without proper attribution, and it is a serious issue in education. AI chatbots can help prevent plagiarism by providing students

with tools and resources that can help them properly cite and reference their sources. For example, an AI chatbot could be programmed to scan a student's paper for common instances of plagiarism, such as copied text or unattributed ideas, and provide suggestions for how to properly attribute the source. In addition, an AI chatbot could provide students with information and guidance on how to avoid plagiarism, such as by providing tips on paraphrasing and summarizing, and by explaining the importance of proper citation and attribution.

However, it is possible that an AI chatbot could cause plagiarism if it is not used responsibly and ethically. For example, if an AI chatbot provides students with information or answers without properly citing the source, it could inadvertently lead students to plagiarize. In addition, if [2] an AI chatbot is programmed to generate text or content without properly attributing the source, it could produce work that is plagiarized. Therefore, it is important for the designers and developers of AI chatbots to carefully consider the potential risks of plagiarism and to take steps to prevent it. This may involve implementing safeguards and protocols [2] to ensure that the information provided by the AI chatbot is properly attributed and that students are encouraged to properly cite and reference their sources. It may also involve educating students and teachers about [6] the importance of academic integrity and the consequences of plagiarism. The potential use of artificial intelligence software by nursing students to write university assessments is a worrying possibility. AI software is designed to mimic human behaviour, but it is not designed to replace human creativity and critical thinking. As such, AI software cannot substitute for the knowledge, skills, and critical thinking that nursing students must develop and demonstrate in their university assessments. [1] The use of AI software could lead to an erosion of the quality of the assessment, with implications for the development of the nursing profession. Furthermore, the use of AI software could lead to a lack of accountability among nursing students, as it is not possible to verify that the work submitted is their own. Finally, the use of AI software could lead to a decrease in the value of the assessments and the overall

quality of the nursing program.

OpenAI [3] is a research institute focused on developing artificial intelligence in a way that is safe and beneficial to humanity. The institute was founded in 2015 by Elon Musk and other prominent tech leaders, and its goal is to advance and promote AI research and development with the aim of creating advanced and intelligent technologies that can help improve the world. OpenAI is known for developing cutting-edge AI technologies and for conducting research [5] in a variety of areas, including natural language processing, computer vision, and robotics. ChatGPT is a large language model trained by OpenAI. It is a machine learning system that [1] is designed to be able to understand and generate natural human language in order to assist with a wide range of tasks, such as answering questions and providing information. It is not a human but is designed [4] to be able to communicate and interact with people in a way that is similar to how a person would. It is constantly learning and improving, and there to help with whatever questions or information you need.

Sources

1	https://techzone090.blogspot.com/2023/01/what-is-artificial-intelligence.html<br>INTERNET<br>7%
2	https://smartclick.ai/articles/what-is-an-ai-chatbot/<br>INTERNET<br>2%
3	https://apps.apple.com/me/app/use-for-openai/id1661181256<br>INTERNET<br>1%
4	https://en.wikipedia.org/wiki/OpenAI#:~:text=OpenAI is an artificial intelligence (AI) research laboratory,a way that benefits humanity as a whole.<br>INTERNET<br>1%
5	https://www.certificationanswers.com/en/your-google-my-business-listing-can-help-customers-reach-you-in-a-variety-of-ways-customers-can-use-a-listed-address-to-visit-your-location-they-can-also-use-a-phone-number-to-place-a-call-which-ot/<br>INTERNET<br>1%
6	https://graduateway.com/the-importance-of-academic-integrity/<br>INTERNET<br>1%

EXCLUDE CUSTOM MATCHES	OFF
EXCLUDE QUOTES	OFF
EXCLUDE BIBLIOGRAPHY	OFF

5.2% PlagScan by Turnitin Results of plagiarism analysis from 2023-01-21 23:10 UTC

ChatGPT

Date: 2023-01-21 23:10 UTC

✱ All sources **3** | Internet sources **3**

☑	[0]	www.igi-global.com/dictionary/artificial-intelligence/1511<br>4.7% 2 matches
☑	[1]	youevolve.net/can-artificial-intelligence-replace-human-intelligence/<br>1.2% 1 matches
☑	[2]	patents.justia.com/patent/10478719<br>0.5% 1 matches

2 pages, 883 words

PlagLevel: 5.2% selected / 5.2% overall

3 matches from 3 sources, of which 3 are online sources.

Settings

Data policy: *Compare with web sources, Check against my documents*

Sensitivity: *Medium*

Bibliography: *Consider text*

Citation detection: *Reduce PlagLevel*

Whitelist: --

[0]Artificial intelligence (AI) is the simulation of human intelligence in machines that are programmed to think and act like humans.[0]These machines are designed to learn, reason, and solve problems in a way that is like human cognition, and they can perform tasks that typically require human intelligence, such as understanding language, recognizing images, and making decisions. The goal of AI research is to create systems that are capable of intelligent behaviour and can be used to improve various aspects of our lives, from healthcare and education to transportation and entertainment. An AI chatbot is a type of chatbot that uses artificial intelligence to enable it to understand and respond to user input in a natural, human-like way. AI chatbots are designed to have conversations with people, using natural language processing to understand and respond to the user's words and intentions.[2]They can be used in a variety of settings, such as customer service, where they can provide answers to common questions and help customers resolve issues, or as personal assistants, where they can help users manage their schedules and tasks. Some AI chatbots are also used in entertainment, such as in video games or as virtual companions.

AI chatbots can be used in education to provide personalized learning experiences for students. For example, an AI chatbot could be used to help students learn a new language by engaging them in conversation and providing feedback and corrections. AI chatbots could also be used to provide tutoring and homework help, by answering questions and providing explanations to help students understand difficult concepts. In addition, AI chatbots could be used to help students stay organized and manage their time, by providing reminders and helping them schedule their tasks and assignments. Overall, AI chatbots have the potential to enhance education by providing personalized, interactive learning experiences for students.

Plagiarism is the act of using someone else's work without proper attribution, and it is a serious issue in education. AI chatbots can help prevent plagiarism by providing students with tools and resources that can help them properly cite and reference their sources. For example, an AI chatbot could be programmed to scan a student's paper for common instances of plagiarism, such as copied text or unattributed ideas, and provide suggestions for how to properly attribute the source. In addition, an AI chatbot could provide students with information and guidance on how to avoid plagiarism, such as by providing tips on paraphrasing and summarizing, and by explaining the importance of proper citation and attribution.

However, it is possible that an AI chatbot could cause plagiarism if it is not used responsibly and ethically. For example, if an AI chatbot provides students with information or answers without properly citing the source, it could inadvertently lead students to plagiarize. In addition, if an AI chatbot is programmed to generate text or content without properly attributing the source, it could produce work that is plagiarized. Therefore, it is important for the designers and developers of AI chatbots to carefully consider the potential risks of plagiarism and to take steps to prevent it. This may involve implementing safeguards and protocols to ensure that the information provided by the AI chatbot is properly attributed and that students are encouraged to properly cite and reference their sources. It may also involve educating students and teachers about the importance of academic integrity and the consequences of plagiarism. The potential use of artificial intelligence software by nursing students to write university assessments is a worrying possibility. AI software is designed to mimic human behaviour, but it is not designed to replace human creativity and critical thinking. As such, AI software cannot substitute for the knowledge, skills, and critical thinking that nursing students must develop and demonstrate in their university assessments. The use of AI software could lead to an erosion of the quality of the assessment, with implications for the development of the nursing profession. Furthermore, the use of AI software could lead to a lack of accountability among nursing students, as it is not possible to verify that the work submitted is their own. Finally, the use of AI software could lead to a

decrease in the value of the assessments and the overall quality of the nursing program.
OpenAI is a research institute focused on developing artificial intelligence in a way that is safe and beneficial to humanity. The institute was founded in 2015 by Elon Musk and other prominent tech leaders, and its goal is to advance and promote AI research and development with the aim of creating advanced and intelligent technologies that can help improve the world. OpenAI is known for developing cutting-edge AI technologies and for conducting research in a variety of areas, including natural language processing, computer vision, and robotics. ChatGPT is a large language model trained by OpenAI. It is a machine learning system that is designed to be able to understand and generate natural human language in order to assist with a wide range of tasks, such as answering questions and providing information. It is not a human but is designed to be able to communicate and interact with people in a way that is similar to how a person would. It is constantly learning and improving, and there to help with whatever questions or information you need.

Welcome to Grammarly Premium!

by Muath

General metrics

5,564	883	35	3 min 31 sec	6 min 47 sec
characters	words	sentences	reading time	speaking time

Score

78

This text scores better than 78% of all texts checked by Grammarly

Writing Issues

59	2	57
Issues left	Critical	Advanced

Plagiarism

9 %

8 sources

9% of your text matches 8 sources on the web or in archives of academic publications

Writing Issues

37	**Clarity**	
11	Wordy sentences	
1	Hard-to-read text	
9	Unclear sentences	
14	Passive voice misuse	
2	Intricate text	
6	**Correctness**	
2	Mixed dialects of english	
4	Punctuation in compound/complex sentences	
12	**Engagement**	
12	Word choice	
4	**Delivery**	
3	Inappropriate colloquialisms	
1	Tone suggestions	

Unique Words

Measures vocabulary diversity by calculating the percentage of words used only once in your document

34%

unique words

Rare Words

36%

Measures depth of vocabulary by identifying words that are not among the 5,000 most common English words.

rare words

Word Length

5.2

Measures average word length

characters per word

Sentence Length

25.2

Measures average sentence length

words per sentence

Welcome to Grammarly Premium!

60 Artificial intelligence (AI) is the simulation of human intelligence in machines
that are programmed[1] to think and act like humans. These machines are
designed to learn, reason, and solve problems in a way that is like human
61 cognition, and they[2] can perform tasks that typically require human intelligence,
such as understanding language, recognizing images, and making decisions.
The goal of AI research is to create systems that are[3] capable of intelligent
62 behaviour[4] and can be used to improve various aspects of our lives, from
healthcare and education to transportation and entertainment. An AI chatbot is
a type of chatbot that uses artificial intelligence to enable it to understand and
respond to user input in a natural, human-like way. AI chatbots are designed[5] to
have conversations[6] with people, using natural language processing to
63 understand and respond to the user's words and intentions. They can be used[7]
in a variety of[8] settings, such as customer service, where they can provide
answers to common questions and help customers resolve issues, or as
personal assistants, where they can help users manage their schedules and
tasks. Some AI chatbots are also used[9] in entertainment, such as in video
games or as virtual companions.

AI chatbots can be used[10] in education to provide personalized learning experiences for students. For example, an AI chatbot could be used[11] to[12] help students learn a new language by engaging them in conversation and providing[illegible] feedback and corrections. AI chatbots could also be used[14] to provide tutoring and homework help,[15] by answering questions and providing explanations to help students understand difficult[16] concepts. In addition, AI chatbots could be used[17] to[18] help students stay organized and manage their time,[19] by providing[20] reminders

and helping them schedule their tasks and assignments. Overall, AI chatbots have the potential to enhance education by providing personalized, interactive learning experiences for students.

64 Plagiarism is the act of[22] using someone else's work without proper attribution, and it[22] is a serious issue in[21] education. AI chatbots can help prevent plagiarism by providing students with tools and resources that can help them properly cite and reference their sources. For example, an AI chatbot could be programmed[23] to scan a student's paper for common instances of plagiarism, such as copied text or unattributed ideas, and provide suggestions for how to properly[24] attribute[25] the source[26]. In addition, an AI chatbot could provide students with information and guidance on how to avoid plagiarism, such as by providing[27] tips on paraphrasing and summarizing,[28] and by explaining the importance of proper citation and attribution.

However, it is possible that[31] an AI chatbot could[29] cause plagiarism if it is not used[30] responsibly and ethically. For example, if an AI chatbot provides students with information or answers without properly citing the source, it could inadvertently lead students to plagiarize. In addition, if an AI chatbot is programmed to generate text or content without properly attributing the source[illegible] , it could produce work that is plagiarized[33,34]. Therefore, it is important[35] for the designers and developers of AI chatbots to carefully consider the potential risks of plagiarism[36] and to[37] take steps to prevent it. This[38] may involve implementing safeguards and protocols to ensure that the information provided by the AI chatbot is properly attributed[39] and that students are encouraged to properly cite and reference their sources[40]. It may also involve

65 educating students and teachers about the importance of academic integrity and the consequences of plagiarism. The potential use of artificial intelligence software by nursing students to write university assessments is a worrying

possibility. AI software is designed[41] to mimic human behaviour[42] [45], but it is not
designed[43,44,45] to replace human creativity and critical thinking. As such, AI software
cannot substitute for the knowledge, skills, and critical thinking that nursing
students must develop and demonstrate in their university assessments. The
use of AI software could lead to an erosion of the quality of the assessment,[47]
with implications for the development of the nursing profession.[46] Furthermore,
the use of[48] AI software could lead to a lack of accountability among nursing
66 students, as it is not possible[48] to verify that the work submitted is their own.
Finally, the use of[49] AI software could lead to a decrease in the value of the
assessments and the overall quality of the nursing program.

67 OpenAI is a research institute focused on developing artificial intelligence in a way that is safe and beneficial to humanity. The institute was founded in 2015 by Elon Musk and other prominent tech leaders, and its[51] goal is to advance and promote AI research and development with the aim of creating[50,51] advanced and intelligent technologies that can help improve the world. OpenAI is known for developing cutting-edge AI technologies and for conducting research in a variety of[52] areas, including natural language processing, computer vision, and robotics. ChatGPT is a large[53] language model trained by OpenAI. It is a machine learning system that is designed[54] to be able[56] to understand and generate natural human language in order to[55,56] assist with a wide range of tasks, such as answering questions and providing information. It is not a human but is designed[57] to be able to[58] communicate and interact with people in a way that is[58]

similar to how a person would. It is constantly learning and improving,[59] and there to help with whatever questions or information you need.

1.	programmed machines	Wordy sentences	Clarity
2.	~~, and they~~ → . They	Hard-to-read text	Clarity
3.	*The goal of AI research is to create systems that are capable of intelligent behaviour and can be used to improve various aspects of our lives, from healthcare and education to transportation and entertainment.*	Unclear sentences	Clarity
4.	~~behaviour~~ → behavior	Mixed dialects of English	Correctness
5.	*are designed*	Passive voice misuse	Clarity
6.	~~have conversations~~ → communicate	Wordy sentences	Clarity
7.	*They can be used*	Passive voice misuse	Clarity
8.	~~a variety of~~ → various	Wordy sentences	Clarity
9.	*are also used*	Passive voice misuse	Clarity
10.	*AI chatbots can be used*	Passive voice misuse	Clarity
11.	*an AI chatbot could be used*	Passive voice misuse	Clarity
12.	~~be used to~~	Wordy sentences	Clarity
13.	~~providing~~ → giving	Word choice	Engagement
14.	*AI chatbots could also be used*	Passive voice misuse	Clarity
15.	help,	Punctuation in compound/complex sentences	Correctness
16.	~~difficult~~ → complex	Word choice	Engagement
17.	*AI chatbots could be used*	Passive voice misuse	Clarity
18.	~~be used to~~	Wordy sentences	Clarity

19.	time,	Punctuation in compound/complex sentences	Correctness
20.	~~providing~~ → offering	Word choice	Engagement
21.	severe issue in, severe issue of	Word choice	Engagement
22.	*Plagiarism is the act of using someone else's work without proper attribution, and it is a serious issue in education.*	Unclear sentences	Clarity
23.	*be programmed*	Passive voice misuse	Clarity
24.	~~properly~~ → correctly	Word choice	Engagement
25.	properly attributing	Wordy sentences	Clarity
26.	to attribute the source properly	Inappropriate colloquialisms	Delivery
27.	~~providing~~ → giving, offering	Word choice	Engagement
28.	summarizing,	Punctuation in compound/complex sentences	Correctness
29.	*it is possible that an AI chatbot could*	Wordy sentences	Clarity
30.	*is not used*	Passive voice misuse	Clarity
31.		Tone suggestions	Delivery
32.	~~source~~ → start, head, original	Word choice	Engagement
33.	plagiarized work	Wordy sentences	Clarity
34.	*is plagiarized*	Passive voice misuse	Clarity
35.	~~important~~ → essential, crucial, vital	Word choice	Engagement
36.	to consider the potential risks of plagiarism carefully	Inappropriate colloquialisms	Delivery

37.	*Therefore, it is important for the designers and developers of AI chatbots to carefully consider the potential risks of plagiarism and to take steps to prevent it.*	Unclear sentences	Clarity
38.	*This*	Intricate text	Clarity
39.	adequately attributed, attributed correctly, appropriately attributed	Word choice	Engagement
40.	to cite and reference their sources properly	Inappropriate colloquialisms	Delivery
41.	*is designed*	Passive voice misuse	Clarity
42.	~~behaviour~~ → behavior	Mixed dialects of English	Correctness
43.	*is not designed*	Passive voice misuse	Clarity
44.	~~designed~~ → intended	Word choice	Engagement
45.	*AI software is designed to mimic human behaviour, but it is not designed to replace human creativity and critical thinking.*	Unclear sentences	Clarity
46.	*The use of AI software could lead to an erosion of the quality of the assessment, with implications for the development of the nursing profession.*	Intricate text	Clarity
47.	~~assessment~~ → evaluation, appraisal	Word choice	Engagement
48.	*Furthermore, the use of AI software could lead to a lack of accountability among nursing students, as it is not possible to verify that the work submitted is their own.*	Unclear sentences	Clarity
49.	~~the use of~~ → using	Wordy sentences	Clarity

50.	to create	Wordy sentences	Clarity
51.	*The institute was founded in 2015 by Elon Musk and other prominent tech leaders, and its goal is to advance and promote AI research and development with the aim of creating advanced and intelligent technologies that can help improve the world.*	Unclear sentences	Clarity
52.	*OpenAI is known for developing cutting-edge AI technologies and for conducting research in a variety of areas, including natural language processing, computer vision, and robotics.*	Unclear sentences	Clarity
53.	~~a large~~ → a significant, an extensive	Word choice	Engagement
54.	*is designed*	Passive voice misuse	Clarity
55.	~~in order to~~ → to	Wordy sentences	Clarity
56.	*It is a machine learning system that is designed to be able to understand and generate natural human language in order to assist with a wide range of tasks, such as answering questions and providing information.*	Unclear sentences	Clarity
57.	*is designed*	Passive voice misuse	Clarity
58.	*It is not a human but is designed to be able to communicate and interact with people in a way that is similar to how a person would.*	Unclear sentences	Clarity
59.	improving,	Punctuation in compound/complex sentences	Correctness
60.	*the simulation of human intelligence in machines that are programmed to think*	ABC Technology - Artificial Intelligence, Blockchain	Originality

		Technology, Cloud Technology for Banking Sector	
61.	*perform tasks that typically require human intelligence, such as understanding language, recognizing images,*	LittleMedia – Medium https://littlemediachannel.medium.com/	Originality
62.	*can be used to improve various aspects of*	Facility, Care Team Promote Healing	Originality
63.	*They can be used in a variety of*	21 Skills That Will Pay You Forever https://www.linkedin.com/pulse/21-skills-pay-you-forever-dr-nicole-gates	Originality
64.	*Plagiarism is the act of using someone else's work without proper attribution, and it*	The Ethics of Content Writing and Avoiding Plagiarism https://www.linkedin.com/pulse/ethics-content-writing-avoiding-plagiarism-ritu-gosavi	Originality
65.	*about the importance of academic integrity and the*	importance of academic integrity https://cchek.libriakmzero.it/page/hmup	Originality
66.	*lead to a decrease in the value of the*	value of the fund - Translation into Italian - examples English ... https://context.reverso.net/translation/english-italian/value+of+the+fund	Originality
67.	*artificial intelligence in a way that is safe and beneficial to*	Switzerland : Data science and artificial intelligence for the public good	Originality
